# Oxidative Stress and Antioxidant Strategies in Cardiovascular Disease

**DOI:** 10.1155/2014/678741

**Published:** 2014-06-17

**Authors:** Adriane Belló-Klein, Neelam Khaper, Susana Llesuy, Dalton Valentim Vassallo, Constantinos Pantos

**Affiliations:** ^1^Physiology Department, Federal University of Rio Grande do Sul, 90050-170 Porto Alegre, RS, Brazil; ^2^Northern Ontario School of Medicine, Lakehead University, Thunder Bay, Canada P7B 5E1; ^3^Faculty of Pharmacy, University of Buenos Aires, Buenos Aires 1121, Argentina; ^4^Santa Casa de Misericórdia School of Medicine (EMESCAM), 29027-502 Vitória, ES, Brazil; ^5^Pharmacology Department, University of Athens, 11527 Athens, Greece

The notion of oxidative stress has changed over the past decades from the idea of being a phenomenon involved exclusively with oxidative damage to a more contemporary concept that includes its role in intracellular signaling pathways. Reactive oxygen species (ROS) such as superoxide anion (O_2^•^_^−^), hydroxyl radical (OH^•^), and hydrogen peroxide (H_2_O_2_) can be generated by different intracellular sources such as NAD(P)H oxidase, xanthine oxidase, myeloperoxidase, and uncoupled nitric oxide synthase [1]. ROS are capable of reacting with several cellular components, resulting in lipid peroxidation, and damage to proteins and DNA. In order to counterregulate these oxidative processes, cells have developed enzymatic and nonenzymatic antioxidant systems that can offer protection by regulating antioxidant response element signaling pathways [2]. In this regard, some phytochemicals, such as sulforaphane, brazilin, chalcone, resveratrol, and curcumin, are reported to modulate translocation and activation of the nuclear factor-erythroid-2-related factor (Nrf2) and regulate antioxidant response [3]. The primary aim of this special issue is to highlight the central role of antioxidants in various experimental models of heart failure and endothelial dysfunction as well as in human studies.

This special issue contains a review article and primary research articles covering a broad range of topics related to the therapeutic potential of antioxidants in heart failure. In a study by Y. Wang et al.,* sulforaphane* attenuation of type-2 diabetes induced aortic fibrosis was associated with the upregulation of Nrf2 expression and function in mice. In another interesting study by M. H. Lee et al.,* resveratrol* inhibited rat aortic vascular smooth muscle cell proliferation, dedifferentiation, and phenotype modulation. This finding was attributed to a differential regulation of prosurvival pathways by resveratrol, reinforcing the protective role of flavonoids by altering the phosphorylation state of some targeted molecules. Another polyphenol covered in this special issue is* methyl gallate* and its ability to afford cardioprotection against cobalt or H_2_O_2_-induced oxidative stress. This polyphenol was able to scavenge ROS, safeguarding mitochondria and cellular DNA and inhibiting the intrinsic apoptotic pathway.

*Apoptosis* is an important element of the cardiac remodeling process [4]. Suppression of apoptosis is a key target in attenuating adverse remodeling process [5]. In a related article featured in this special issue, a* microRNA* (miRNA) that targets glutathione peroxidase was utilized to explore its potential cardioprotective role against oxidative stress-induced apoptosis. This miRNA was markedly upregulated in apoptotic cells and its downregulation reduced the mitochondrial apoptotic pathway in cardiomyocytes exposed to oxidative stress. These novel findings may have some therapeutic implications for a variety of cardiovascular diseases related to ROS, including atherosclerosis.

The role of antioxidants in* atherosclerosis* was another key topic explored in this special issue. An elegant paper by A. J. Lepedda et al. demonstrated that the prooxidant environment present in atherosclerotic plaque may oxidatively modify filtered albumin and the contribution of glutathione in maintaining the* intraplaque thiols equilibrium*. Another interesting study by M. Macharia et al. featured in this issue evaluated the association of indices of* paraoxonase*, an enzyme that prevents the oxidation of LDL cholesterol, as well as the oxidative status with subclinical cardiovascular disease in mixed-ancestry South Africans. Diabetic subjects of this population displayed a significant decrease in paraoxonase and antioxidants as well as an increase in oxidized LDL and lipid peroxidation. Carotid intima-media thickness of these patients was negatively correlated with indices of antioxidant activity and positively correlated with measures of lipid oxidation. E. Tuncay et al. also explored the role of antioxidants in diabetes. The authors elegantly demonstrated that an enhancement of antioxidant defense in diabetics prevented diastolic dysfunction due to modulation of the ryanodine receptor, leading to normalized intracellular concentrations of calcium and zinc in cardiomyocytes.

Estrogen therapy as another antioxidant strategy is explored by two articles featured in this issue. The influence of estrogen on* coronary resistance* was studied by P. C. Schenkel et al. where they investigated the modulatory role of nitric oxide and H_2_O_2_ levels in female rats. The data suggest that, in the absence of estrogen, coronary resistance regulation seems to be more dependent on H_2_O_2_ which is maintained at low levels by increased catalase activity. The data provides a new insight regarding the role of oxidative stress balance in the regulation of coronary tone. One compelling question that is always raised regarding estrogen therapy is the optimal dose that should be used. An interesting paper by C. Campos et al. indicates that a* low dose of estrogen* (40% less than the pharmacological dose) was just as effective as a high dose for promoting improvement in cardiovascular function and reducing oxidative stress, thereby supporting the approach of using low dose of estrogen in clinical settings to minimize the risks associated with estrogen therapy.

The cardioprotective role of* thyroid hormones* has also been featured in this special issue. Administration of this hormone has been associated with increased ROS, Nrf2, thioredoxin, and heme-oxygenase levels in cardiac tissue [6]. It seems that a thyroid hormone-dependent counterregulatory response could represent a hormetic effect, in order to stabilize redox environment and provide cell survival [7]. An elegant review by C. Pantos and I. Mourouzis highlights the role of thyroid hormones in ischemia/reperfusion injury and the conversion from pathologic to physiologic growth after myocardial infarction via TR*α*1 receptor.

In summary, this special issue covers a wide range of topics addressing the role of oxidative stress and antioxidants in the pathophysiology of heart failure. These articles not only enrich our understanding of how oxidative stress plays an important role in heart failure but also provide evidence on antioxidant therapies in this condition.
